# Segmental versus extended colectomy for colonic Crohn’s disease in advanced therapy era: a systematic review and Bayesian meta-analysis

**DOI:** 10.1007/s10151-026-03355-z

**Published:** 2026-06-16

**Authors:** L. M. Delgado, B. F. Pompeu, L. M. Peñaranda, G. H. A. Martins, P. Viana, C. Theis, S. M. P. de Figueiredo, F. B. Formiga

**Affiliations:** 1 Faculdade de Medicina da Universidade Federal de Minas Gerais, Av. Prof. Alfredo Balena, 190 - Santa Efigênia, Belo Horizonte, MG 30130-100 Brazil; 2 Department of Colorectal Surgery, Heliópolis Hospital, São Paulo, SP Brazil; 3 Universidade Municipal de São Caetano do Sul (USCS), São Paulo, SP Brazil; 4 Extreme South, Criciúma, Brazil; 5 Department of Surgery, University of North Carolina, Chapel Hill, NC USA

**Keywords:** Crohn’s disease, Colonic Crohn’s, Colectomy, Surgical recurrence, Meta-analysis

## Abstract

**Background:**

The optimal extent of resection for colonic Crohn’s disease (CD) remains controversial. Segmental colectomy (SC) may preserve bowel and avoid permanent stoma but has been historically associated with higher recurrence than extended colectomy (EC). In the advanced therapy era, contemporary data and Bayesian methods may refine this risk–benefit balance.

**Methods:**

PubMed, Embase, and Cochrane Library were searched from inception to December 1, 2025. The primary outcome was surgical recurrence; secondary outcomes were reoperation, permanent stoma, overall postoperative complications, and mortality. Frequentist random-effects models (restricted maximum likelihood) were used to pool odds ratios (ORs) with 95% confidence intervals (CIs), with heterogeneity assessed by *I*^2^ and leave-one-out analyses. Bayesian random-effects meta-analyses (bayesmeta) were performed for surgical recurrence, reoperation, and permanent stoma using vague priors and empiric Turner priors for heterogeneity, with additional models incorporating historical evidence from the advanced therapy era.

**Results:**

Five observational studies (1095 patients; 501 SC, 594 EC) were included. Surgical recurrence did not differ significantly between SC and EC (OR 1.15; 95% CI 0.53–2.50; *I*^2^ = 74%). Reoperation (OR 1.36; 95% CI 0.71–2.61; *I*^2^ = 68%), permanent stoma (OR 0.22; 95% CI 0.03–1.39; *I*^2^ = 87%), and overall complications (OR 0.75; 95% CI 0.40–1.40; *I*^2^ = 64%) were also not significantly different. Mortality was reported in one study (1 SC vs 5 EC). Bayesian models suggested a higher probability of surgical recurrence and reoperation after SC but with wide credible intervals and substantial heterogeneity, whereas SC showed a consistently high probability of reducing permanent stoma risk (posterior OR ~ 0.23; *P*[OR ≤ 1] > 0.95 in primary models; reinforced by historical priors). Leave-one-out analyses identified influential studies for some outcomes but did not materially alter the overall interpretation. All studies had a moderate risk of bias, mainly due to confounding and selection.

**Conclusion:**

In contemporary observational evidence, SC and EC show broadly comparable outcomes for surgical recurrence, reoperation, complications, and mortality, while SC is strongly favored with respect to permanent stoma avoidance. Operative decision-making should weigh a probable increase in recurrence risk after SC against the clinically meaningful functional benefit of stoma preservation, recognizing the limitations imposed by observational designs, heterogeneity, and sparse data.

**Supplementary Information:**

The online version contains supplementary material available at 10.1007/s10151-026-03355-z.

## Introduction

Crohn’s disease (CD) is a chronic inflammatory bowel disease (IBD) with a relapsing–remitting course, leading to progressive gastrointestinal damage and significant disability over time [1]. CD can affect any part of the GI tract, most commonly the terminal ileum and proximal colon, in a segmental, transmural pattern. Its progressive nature may result in complications such as fistulas, abscesses, and strictures, frequently necessitating surgical intervention [2, 3]. CD can also affect the large intestine and rectum, with surgical management guided by disease extent, pattern, perianal involvement, and presence of neoplasia.

Historically, segmental colectomy (SC), usually indicated for disease limited to segments of the colon, has been associated with higher postoperative complication rates, whereas subtotal or total colectomy, often indicated for extensive colonic involvement or when terminal ileum involvement is present, has typically demonstrated longer disease-free intervals. Recent evidence, however, suggests that in carefully selected patients, SC does not necessarily increase the risk of surgical recurrence, especially in the current era of advanced therapies [4]. Previous meta-analyses indicated that SC was linked with a higher risk of postoperative complications but a lower likelihood of permanent stoma formation compared to extended colectomy (EC), with both approaches showing similar overall and surgical recurrence rates [5].

However, these analyses included many cases from before the advanced therapies and do not capture emergent evidence [6–9], making an updated systematic review evaluation necessary to understand the current situation. Furthermore, given the limited number of contemporary studies and the substantial heterogeneity observed across published estimates, there is a growing need for complementary analytical frameworks such as Bayesian model approaches, which allow incorporation of prior information and provide more robust inference in settings with sparse or inconsistent evidence. Therefore, this systematic review and meta-analysis aims to assess and compare the efficacy and safety of SC and EC in the surgical management of colonic CD.

## Methods

### Protocol and registration

We performed a systematic review and meta-analysis according to the Cochrane Handbook for Systematic Reviews of Interventions and structured it according to the Preferred Reporting Items for Systematic Reviews and Meta-Analysis (PRISMA) guidelines, presented in Table S1 (Supplementary Material 1) [10, 11]. The study protocol was registered in the International Prospective Register of Systematic Reviews (PROSPERO) under registration number CRD420251244847 [12].

### Eligibility criteria

Inclusion in this meta-analysis was restricted to studies meeting all of the following eligibility criteria: (1) adult patients (≥ 18 years old) diagnosed with colonic CD; (2) studies directly comparing SC with EC (including subtotal, total, and proctocolectomy procedures) for the treatment of colonic CD; (3) reporting at least one outcome of interest; and (4) studies conducted in the biologic therapy era (2010 onwards), when the use of biologic agents had become part of standard medical management for CD. Studies were excluded if they did not meet the inclusion criteria or if they were single-arm studies, case reports, trial registrations without results, meta-analyses, reviews, or animal studies.

### Outcomes

The primary outcome was (1) the rate of surgical recurrence, defined as reoperation due to disease recurrence. The secondary outcomes were (2) reoperation, defined as any subsequent surgical procedure; (3) permanent stoma; (4) overall complications; and (5) postoperative mortality.

### Search strategy and study selection

We systematically searched PubMed, Embase, and Cochrane Library databases from inception to December 1, 2025. The search strategy was (“Crohn Disease” OR “Crohn Colitis”) AND (“Segmental Colectomy” OR “Segmental Resection” OR “Hemicolectomy”) AND (“Subtotal Colectomy” OR “Total Colectomy” OR “Proctocolectomy” OR “Complete Colectomy”). We also searched the references of the included studies and previous systematic reviews and meta-analyses aiming for the inclusion of additional studies [13].

Two authors (L.M.P. and L.M.D.) independently conducted the search, imported results into Rayyan Software (Qatar Computing Research Institute, Qatar Foundation), and triaged the studies. After the exclusion of duplicates and titles/abstracts unrelated to the clinical question, the eligibility of each remaining study was assessed based on the review of the full-text articles. Disagreements were solved by a third author (B.F.P.).

### Data extraction

Two authors (L.M.P. and G.H.A.M.) independently extracted data from the included studies using a standardized form. Extracted variables included general study details (title, authors, country, year of publication, study design, and study period), sample size, patient characteristics (age, sex, and history of previous procedures for CD), follow-up duration, surgical indications, operative details, disease extent as reported in the original studies, including the Montreal classification [14], number of involved colonic segments, and extent of colitis, surveillance strategy and the predefined clinical outcomes of interest. The extracted raw outcome data used for the analyses are provided in Tables S1–S5 (Supplementary Material 2).

### Risk of bias assessment

Two independent reviewers (L.M.D. and L.M.P.) assessed the Risk of Bias in Non-Randomized Studies of Interventions (ROBINS-I V2) tool [15]. Disagreements were resolved through consensus. Publication bias could not be assessed adequately because the power of this test is insufficient to discriminate between chance and true funnel plot asymmetry when analyzing fewer than 10 studies [16].

### Statistical analysis

#### Frequentist analysis

Odds ratios (ORs) with 95% confidence intervals (CIs) were pooled for binary outcomes. We deemed a *P* value of < 0.05 as statistically significant for overall effect estimates. Restricted maximum likelihood random-effects models were used for all outcomes. Heterogeneity was assessed using Cochran *Q* test and *I*^2^ statistics; *P* values < 0.10 and *I*^2^ > 25% were considered to indicate significant heterogeneity. For outcomes with significant heterogeneity, we performed leave-one-out sensitivity analysis to identify influential studies and their effect on the pooled estimates. All the statistical analyses were conducted using R statistical software (version 4.4.2; R Foundation for Statistical Computing, Vienna, Austria).

#### Bayesian analysis

We performed a Bayesian random-effects meta-analysis to estimate the posterior distribution of the overall effect (*μ*) and the between-study heterogeneity (*τ*). The Bayesian framework updates prior assumptions with the observed data, generating posterior distributions that reflect both sources of uncertainty [17]. All analyses were conducted using the bayesmeta package in R [18].

#### Prior specification

For the three outcomes evaluated in this review (surgical recurrence, reoperation, and permanent stoma), the primary analysis employed a vague prior for the overall effect (*μ* ~ Normal(0, 2.7^2^)), consistent with a non-informative prior on the treatment direction. For the heterogeneity parameter, we used the empirically derived Turner et al. log-Normal prior, which is recommended for surgical and device-related outcomes and is applicable to non-pharmacological comparators [19].

Because surgical recurrence and permanent stoma have prior evidence from meta-analyses conducted in the advanced therapy era [5], we performed an additional model using a historical prior for *μ*. For surgical recurrence, *μ* was centered at the Angriman estimate (*μ* ~ Normal(− 0.65, 0.20^2^)). For a permanent stoma, the historical distribution was centered at *μ* ~ Normal(− 0.65, 0.20^2^). These models evaluate the impact of incorporating external evidence from the advanced therapy era on posterior estimates [5].

To assess robustness, two alternative heterogeneity priors were fitted: a weakly informative Half-Normal(0.5) and a more permissive Half-Normal(1.5). These priors represent increasingly flexible assumptions about between-study variability [18].

#### Posterior inference

Posterior summaries were reported as means and 95% highest-density credible intervals (CrI), defined as the smallest interval containing 95% of the posterior density [20]. In addition to effect estimates, we computed clinically interpretable posterior probabilities, including the probability that SC is beneficial (*P*(OR ≤ 1)), the probability of clinically meaningful benefit (*P*(OR ≤ 0.75)), and the probability of clinically relevant harm (*P*(OR ≥ 1.5)) [17]. Finally, the posterior predictive distribution was obtained to estimate the range of treatment effects expected in future exchangeable populations, incorporating uncertainty in both *μ* and *τ* [21].

## Results

### Study selection and characteristics

**Fig. 1:**
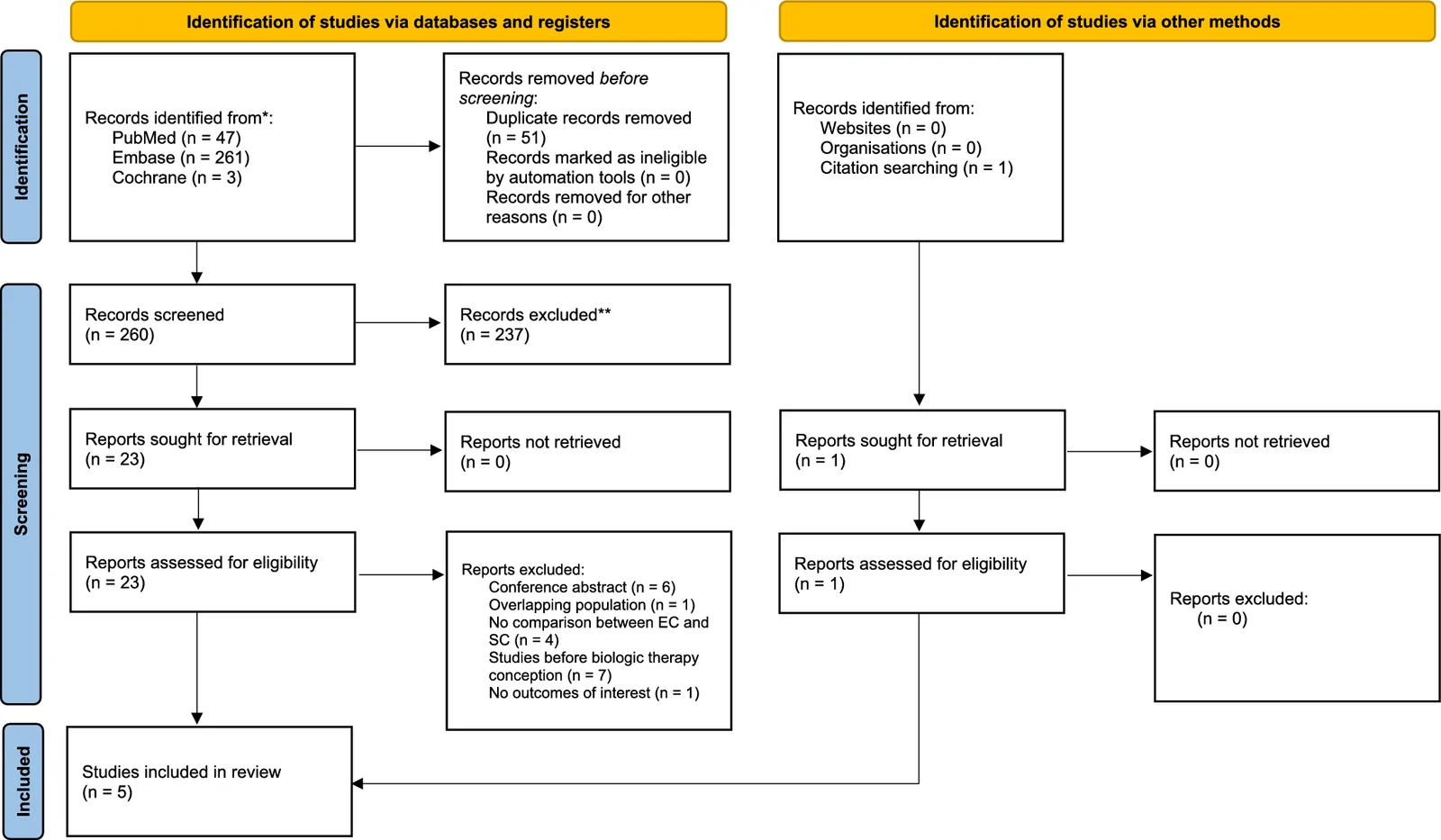
Preferred Reporting Items for Systematic Reviews and Meta-Analysis (PRISMA) flow diagram of study screening and selection

As detailed in Fig. 1, the initial search identified 311 records. After the removal of duplicate records and screening based on title and abstract, 237 studies were excluded for not meeting the predefined inclusion criteria. Twenty-four studies remained for full-text review according to the prespecified criteria. Of these, five observational studies were ultimately included [6–9, 22]. Nineteen studies were excluded after full-text review. These excluded studies, along with their specific references and reasons for exclusion, are listed in Table S2 (Supplementary Material 1). The cohort comprised 1095 patients, of whom 501 (45.8%) underwent SC and 594 (54.2%) underwent EC. The baseline characteristics are detailed in Table 1.

**Table 1:** Baseline characteristics of the included studies

First author, year	Country	Design	Study period	No. of patients		Mean age, years		Male, %		Patients undergoing first operation for cCD, %		Mean follow-up, years		Indications for operation, %
				SC	EC	SC	EC	SC	EC	SC	EC	SC	EC	
Celentano, 2021	Italy	Multicenter Retrospective	2018–2019	30	55	47*	39*	46.6	41.8	50	56.4	30 days		NA
Kiran, 2011	USA	Single center Retrospective	1995–2009	49	59	38.8	41.5	47	32	100	100	5.6*^§^		Stricture disease, 27 Penetrating disease, 31 Inflammatory disease, 42
Lee 2016	Korea	Single center Retrospective	1997–2011	71	45	23	21	71.8	48.9	100	100	7.9	8.5	NA
Pellino, 2022	Italy, Spain, France and UK	Multicenter Prospective	2000–2019	285	402	NA	NA	47.7	41	100	100	7.1^§^		Stenosing disease, 48.62 Perforative disease, 19.80 Refractory disease, 31.59
Sensi, 2022	USA and Europe	Multicenter Retrospective	2010–2020	66	33	59^a^	54^a^	62.12	63.64	71.21	54.55	3.58		Colorectal cancer, 100

*cCD* colonic Crohn’s disease, *EC* extended colectomy, *SC* segmental colectomy, *NA* not available

*Median

^§^The value represents the combined data of both groups

^a^The value represents the mean age at the time of surgery

Disease extent was reported heterogeneously across studies using different classification systems. Overall, patients undergoing EC tended to have more extensive disease involvement compared with those undergoing SC. The distribution of disease extent across studies is summarized in Table 2.

**Table 2:** Extent of disease in the included studies

First author, year	Classification/definition	No. of patients		Segmental colectomy^a^	Extended colectomy^a^
		SC	EC		
Celentano, 2021	Montreal classification	30	55	L2: 19 (63.3) L3: 11 (36.7)	L2: 30 (54.5) L3: 25 (45.5)
Kiran, 2011	Number of involved colonic segments	49	59	Single segment: 33 (67.3) Two segments: 16 (32.7)	Single segment: 31 (52.5) Two segments: 28 (47.5)
Lee, 2016	Number of involved segments	71	45	Single segment: 56 (78.9) ≥ 2 segments: 15 (21.1)	≥ 2 segments: 45 (100)
Pellino, 2022	Montreal classification	285	402	L2: 143 (50.2) L3: 142 (49.8) L4 involvement: 11 (3.9)	L2: 277 (68.9) L3: 125 (31.1) L4 involvement: 11 (2.7)
Sensi, 2022	Extent of colitis	66	33	No colitis: 30 (45.5) Segmental colitis/proctitis: 28 (42.4) Pancolitis/proctocolitis: 8 (12.1)	No colitis: 6 (18.2) Segmental colitis/proctitis: 11 (33.3) Pancolitis/proctocolitis: 16 (48.5)

*SC* segmental colectomy, *EC* extended colectomy, *L2* colonic Crohn’s disease, *L3* ileocolonic Crohn’s disease, *L4* upper gastrointestinal involvement according to the Montreal classification, *GI* gastrointestinal

^a^Data are reported as * n* (%)

Preoperative medical therapy is summarized in Table 3. Reporting of medical treatment varied across studies. The study by Kiran et al. did not report information on preoperative medical therapy [22]. Overall, steroids, immunomodulators, and anti-TNF agents were the most commonly reported therapies across studies. In contrast, information on anti-integrin α4β7 therapy and combined therapy was limited and was reported only in the study by Pellino et al. [8].

**Table 3:** Baseline preoperative medical therapy of patients included in the studies

First author, year	No. of patients		Steroids^a^		Immunomodulators^a^		Anti-TNF^a^		5-ASA^a^		Anti-integrin α4β7^a^	
	SC	EC	SC	EC	SC	EC	SC	EC	SC	EC	SC	EC
Celentano, 2021	30	67	11 (36.6)	31 (46.3)	3 (10)	9 (13.4)	9 (30)	16 (23.9)	NA	NA	NA	NA
Kiran, 2011	49	59	NA	NA	NA	NA	NA	NA	NA	NA	NA	NA
Lee, 2016	71	45	50 (70.4)	34 (75.6)	38 (53.5)	22 (48.9)	9 (12.7)	10 (22.2)	65 (91.5)	42 (93.3)	NA	NA
Pellino, 2022	285	402	44 (15.4)	89 (22.1)	47 (16.5)	35 (8.7)	37 (13)	58 (14.4)	88 (30.9)	103 (25.6)	11 (3.8)	11 (2.8)
Sensi, 2022	66	33	9 (13.6)	3 (9.0)	NA	NA	11 (16.7)	6 (18.2)	NA	NA	NA	NA

^a^Data are reported as * n* (%)

*SC* segmental colectomy, *EC* extended colectomy, *TNF* tumor necrosis factor, *5-ASA* 5-aminosalicylic acid, *NA* not available

### Frequentist meta-analysis of perioperative outcomes

#### Primary outcome

**Fig. 2:**
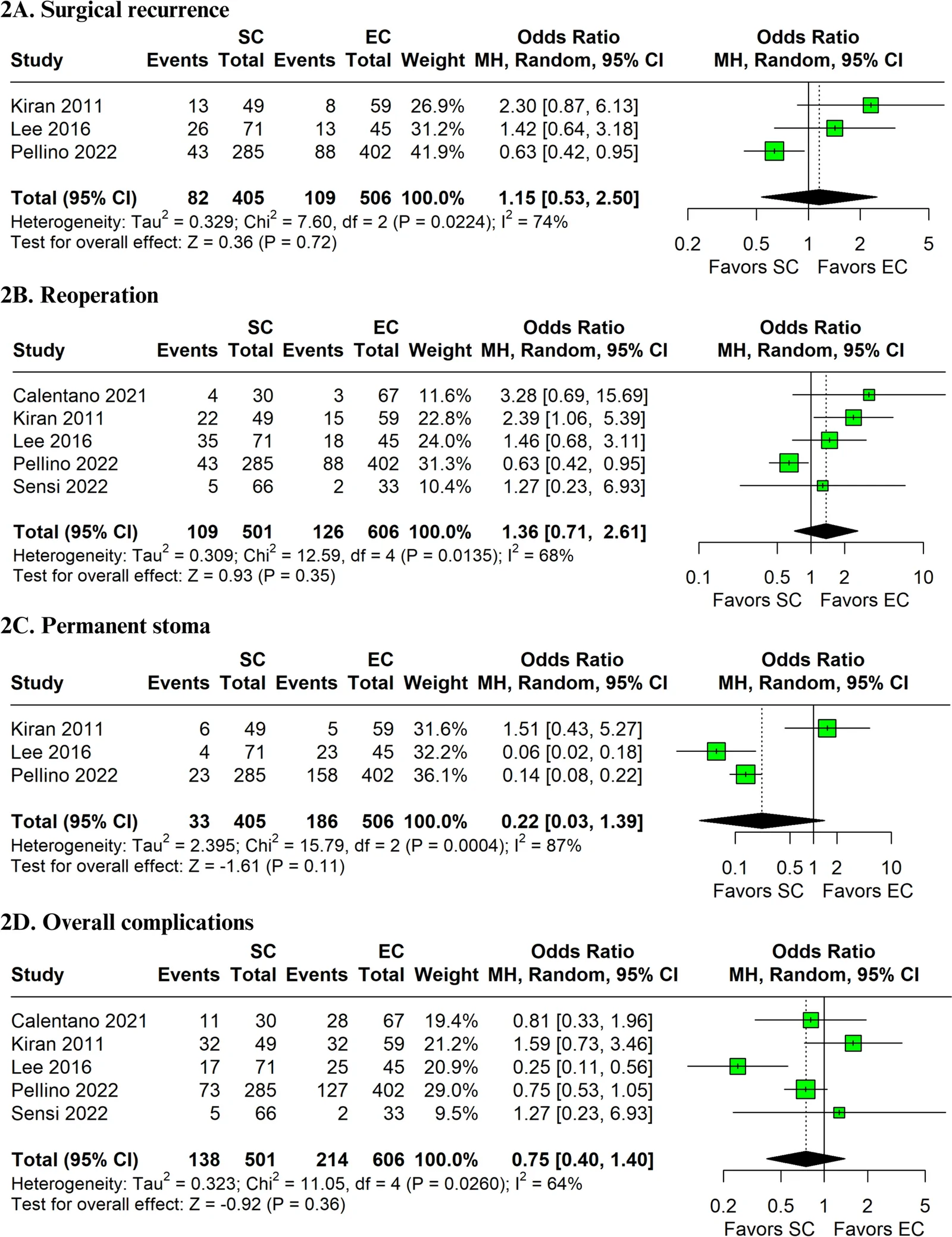
Frequentist meta-analysis of surgical outcomes: **a** surgical recurrence, **b** reoperation, **c** permanent stoma, **d** overall postoperative complications

Three studies (Kiran et al., Lee et al., and Pellino et al.) contributed data to the analysis of surgical recurrence [8, 9, 22]. Lee et al. reported a median follow-up of 5.6 years and recurrence rates at 5 years, while Pellino et al. reported recurrence at 5, 10, and 15 years. To ensure comparability across studies, recurrence rates were extracted and analyzed at the 5-year follow-up time point, which was the most consistently reported interval across studies. A total of 911 patients were included in the analysis of surgical recurrence, with 405 in the SC group and 506 in the EC group. Surgical recurrence occurred in 82 patients (20.2%) in the SC group and 109 patients (21.5%) in the EC group. There were no significant differences between groups in terms of surgical recurrence (OR 1.15 95% CI 0.53–2.50; *P* = 0.72; Cochran’s *Q* = 7.60, df = 2, *P* = 0.022; *I*^2^ = 74%; Fig. 2a).

#### Secondary outcomes

For reoperation, although not statistically significant (OR 1.36; 95% CI 0.71–2.61; *P* = 0.35; Cochran’s *Q* = 12.59, df = 4, *P* = 0.0135; *I*^2^ = 68%; Fig. 2b), rates were higher in EC (20.8%; 126/606) than SC (21.8%; 109/501). Permanent stoma was also more frequent with EC (36.8%; 186/506) than SC (8.1%; 33/405), though this difference was not statistically significant (OR 0.22; 95% CI 0.03–1.39; *P* = 0.11; Cochran’s *Q* = 15.79, df = 2, *P* = 0.0004; *I*^2^ = 87%; Fig. 2c). Overall complications occurred in 27.5% (138/501) of SC patients versus 35.3% (214/606) of EC patients, without a significant difference between groups (OR 0.75; 95% CI 0.40–1.40; *P* = 0.36; Cochran’s *Q* = 11.05, df = 4, *P* = 0.026; *I*^2^ = 64%; Fig. 2d). Only Pellino et al. reported mortality, with one event in the SC group and five in the EC group [8].

### Bayesian meta-analysis of perioperative outcomes

#### Surgical recurrence

**Fig. 3:**
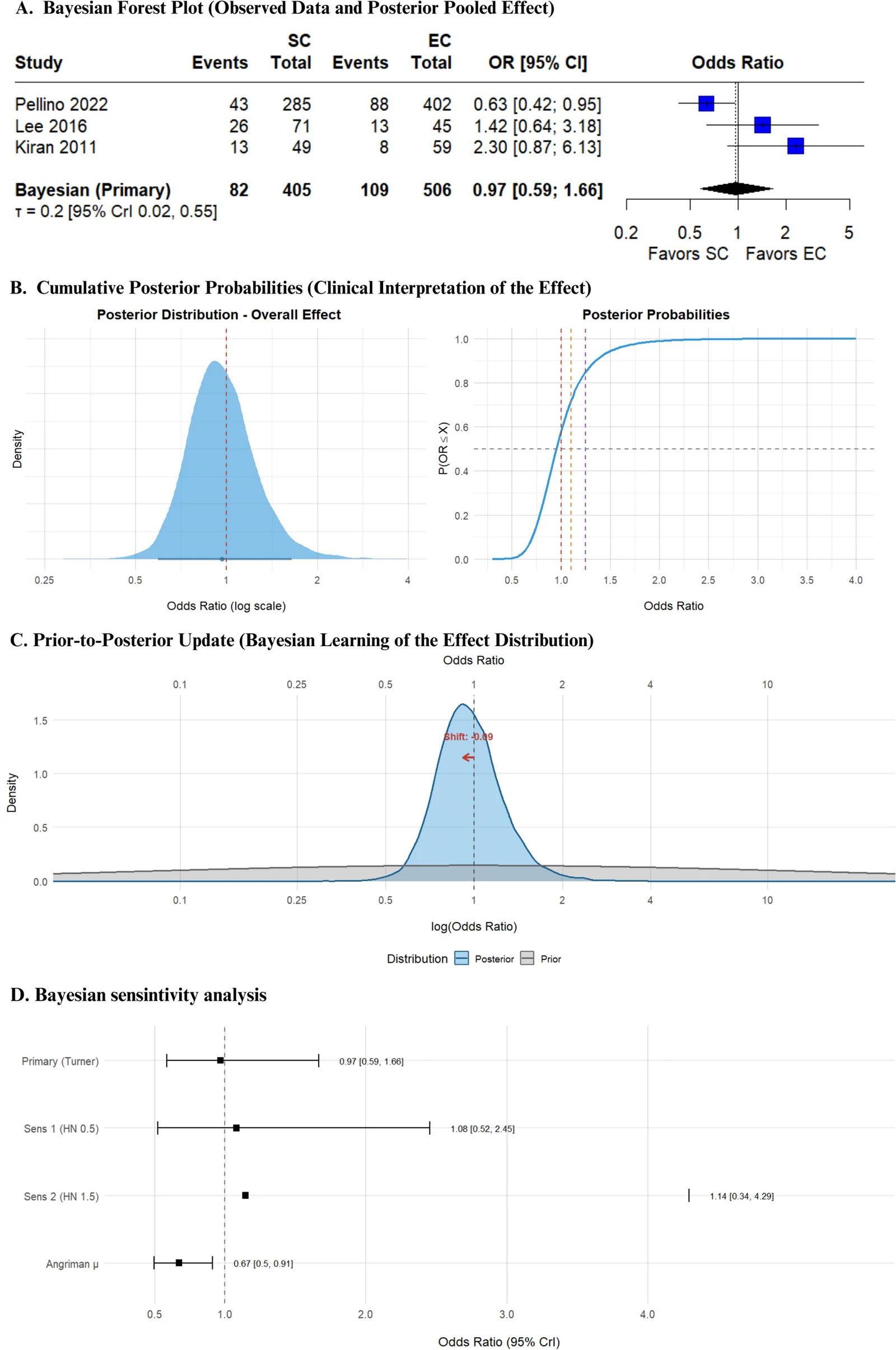
Bayesian meta-analysis of surgical recurrence: **a** Bayesian forest plot (observed data and posterior pooled effect), **b** cumulative posterior probabilities (clinical interpretation of the effect), **c** prior to posterior update (Bayesian learning of the effect distribution), **d** Bayesian sensitivity analysis

The Bayesian primary model (vague *μ* prior + Turner prior for *τ*) resulted in a posterior OR of 1.10 (95% CrI 0.48–2.75), with a 42.5% probability that SC reduces recurrence (*P*[OR ≤ 1]) and a 57.5% probability of higher recurrence compared with EC (*P*[OR > 1]) (Fig. 3a, b). Updating prior evidence with the historical* μ* informed by data from the advanced therapy era shifted the posterior toward a lower recurrence risk with SC (posterior OR 0.62, 95% CrI 0.42–0.89) and increased the probability of benefit to 99.7% (Fig. 3c). Sensitivity analyses using weakly informative and more permissive heterogeneity priors yielded similar results (posterior OR 1.08–1.14), confirming the robustness of the findings (Fig. 3d). In the primary model and heterogeneity sensitivity analyses, Bayesian inference indicated a slightly higher probability of recurrence after SC, although uncertainty remained substantial due to the limited number of studies. Full numerical results, posterior probability classifications, and prior-to-posterior summaries are provided in Fig. S1 and Tables S3–S5 (Supplementary Material 1).

#### Reoperation

**Fig. 4:**
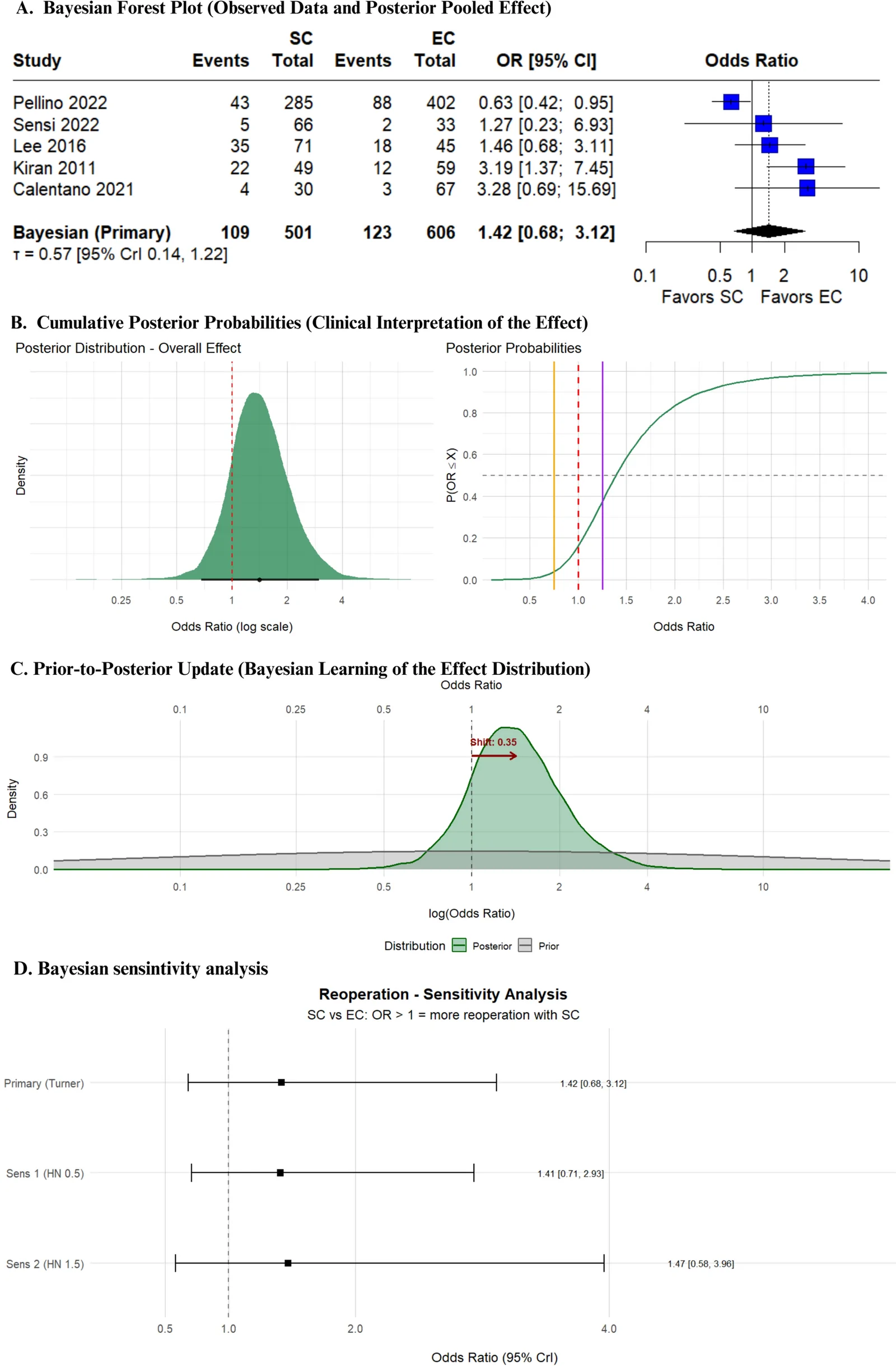
Bayesian meta-analysis of reoperation: **a** Bayesian forest plot (observed data and posterior pooled effect), **b** cumulative posterior probabilities (clinical interpretation of the effect), **c** prior to posterior update (Bayesian learning of the effect distribution), **d** Bayesian sensitivity analysis

The Bayesian primary model (vague *μ* prior + Turner prior for *τ*) resulted in a posterior OR of 1.42 (95% CrI 0.68–3.12), with a 16.1% probability that SC reduces reoperation (*P*[OR ≤ 1]) and an 83.9% probability of higher reoperation compared with EC (*P*[OR > 1]) (Fig. 4a, b). Updating prior uncertainty through the prior-to-posterior learning step showed only a minimal shift in the effect distribution, reflecting limited information from the available evidence (Fig. 4c). Sensitivity analyses using weakly informative and more permissive heterogeneity priors produced highly consistent results (posterior OR 1.41–1.47), confirming that conclusions were robust across different prior assumptions (Fig. 4d). Across all priors, Bayesian inference indicated a high probability of higher reoperation rates following SC, although uncertainty remained substantial due to the small number of included studies. Full posterior estimates, probability classifications, and sensitivity results are provided in Fig. S2 and Tables S6–S8 (Supplementary Material 1).

#### Permanent stoma

**Fig. 5:**
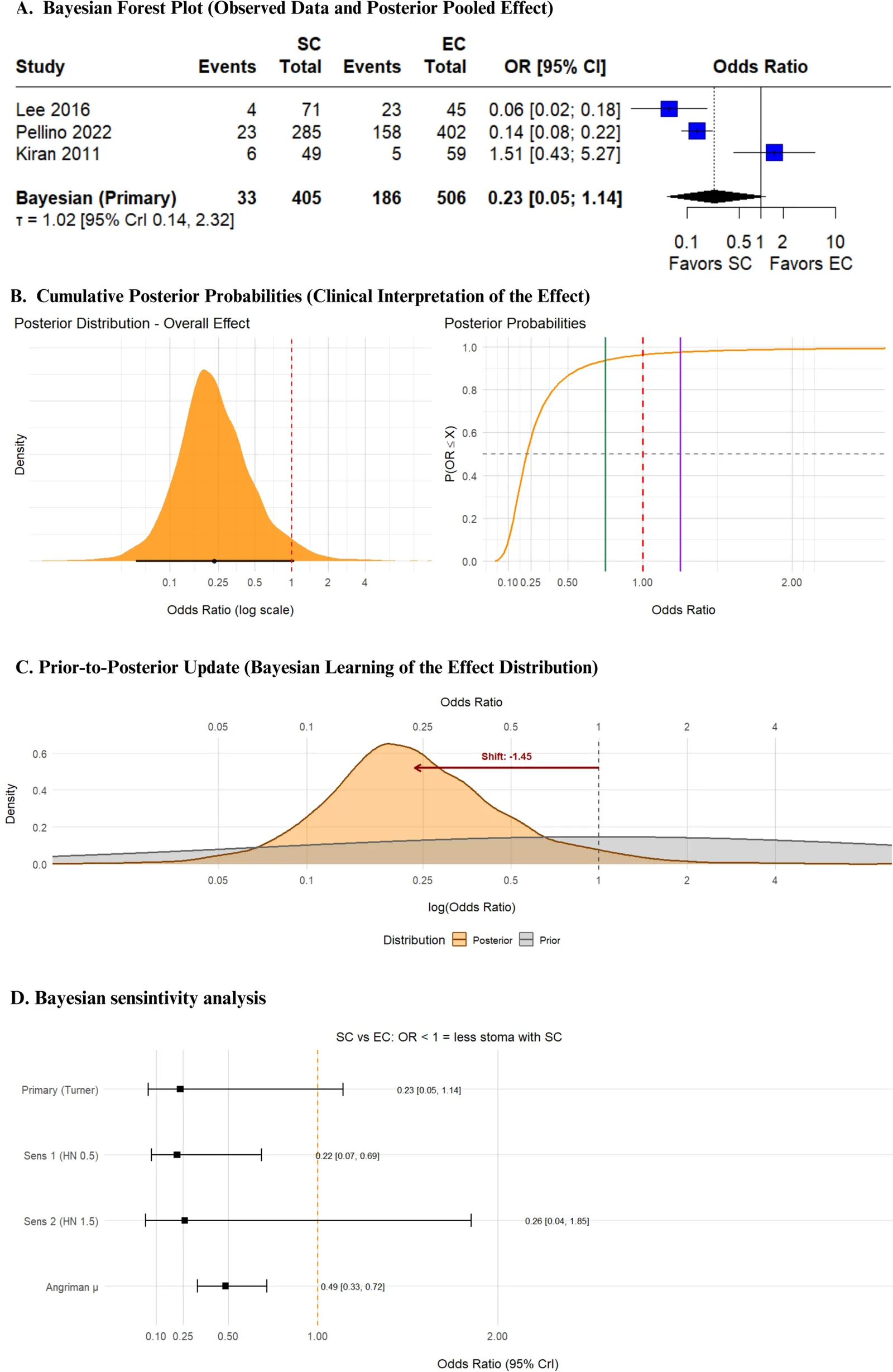
Bayesian meta-analysis of permanent stoma: **a** Bayesian forest plot (observed data and posterior pooled effect), **b** cumulative posterior probabilities (clinical interpretation of the effect), **c** prior to posterior update (Bayesian learning of the effect distribution), **d** Bayesian sensitivity analysis

The Bayesian primary model (vague *μ* prior + Turner prior for *τ*) resulted in a posterior OR of 0.23 (95% CrI 0.05–1.14), with a 96.4% probability that SC reduces the risk of permanent stoma (*P*[OR ≤ 1]) and a 3.6% probability favoring EC (*P*[OR > 1]) (Fig. 5a, b). Updating prior evidence through the prior-to-posterior learning step demonstrated a substantial shift toward stronger protection with SC, consistent with the magnitude of the posterior effect (Fig. 5c). Sensitivity analyses using weakly informative and more permissive heterogeneity priors yielded posterior ORs ranging from 0.22 to 0.26, with *P*(OR ≤ 1) remaining above 92% in all models, confirming the robustness of the findings (Fig. 5d). Across all priors, Bayesian inference provided strong to very strong evidence that SC reduces the likelihood of permanent stoma. The historical* μ* prior further reinforced this conclusion, producing a posterior OR of 0.49 (95% CrI 0.33–0.72) with a 100% probability of benefit. Full posterior estimands, probability classifications, and sensitivity results are reported in Fig. S3 and Tables S9–S11 (Supplementary Material 1).

#### Frequentist sensitivity analysis

The results of the leave-one-out sensitivity analysis are presented in Figs. S4–S7 (Supplementary Material 1). For surgical recurrence and reoperation, exclusion of the study by Pellino et al. eliminated heterogeneity while maintaining a nonsignificant result. For permanent stoma, exclusion of the study by Kiran et al. shifted the result from nonsignificant to significant in favor of SC. For overall complications, exclusion of the study by Lee et al. markedly reduced heterogeneity while the result remained nonsignificant.

#### Risk of bias assessment

Table S12 (Supplementary Material 1) summarizes the ROBINS-I V2 assessment, showing an overall moderate risk of bias, mainly from confounding, selection bias, deviations from intended interventions, and variability in outcome measurement. All studies were non-randomized, with treatment choice influenced by disease severity and surgeon preference, and with exclusions of emergencies, prior resections, or specific CD phenotypes, which limited generalizability and left residual confounding. Postoperative management (immunosuppressants, biologics, and recurrence surveillance) and follow-up duration varied across studies, affecting comparability of recurrence and survival outcomes. Missing data (with 7–10% of patients excluded) and heterogeneous methods to ascertain recurrence (clinical, imaging, or endoscopy) further contributed to attrition and detection bias.

## Discussion

In this systematic review and meta-analysis of five observational studies comparing SC and EC for colonic CD, we observed distinct patterns across the main clinical outcomes. While the frequentist analyses suggested that the two techniques are broadly comparable, the Bayesian analyses suggested a higher probability of surgical recurrence and reoperation after SC, although these estimates were imprecise and limited by substantial heterogeneity. In contrast, SC consistently showed a high probability of reducing the risk of permanent stoma formation, which emerged as its most robust clinical advantage. The direction and magnitude of these findings were concordant across analytical frameworks and remained stable in sensitivity analyses, suggesting that operative decision-making should balance the increased likelihood of recurrence against the meaningful functional benefit of stoma preservation.

Elective colectomy, when performed in carefully selected patients, offers effective symptom control, improved quality of life, and mitigation of long-term complication, all meaningful outcomes given the heterogeneous presentation of colonic CD [23, 24]. The therapeutic sequence in managing colonic CD typically begins with aggressive medical therapies, including corticosteroids, immunomodulators, biologic agents, or small molecules [25]; however, surgical intervention becomes necessary for a subset of patients when these treatments fail the patient. The decision-making framework guiding surgical resection thus involves careful assessment of disease extent and severity, as well as thorough evaluation of prior medical management strategies, particularly given the increasing role of biologic therapies in recent years [26, 27].

A meta-analysis by Angriman et al. demonstrated no significant differences between SC and subtotal colectomy regarding overall and surgical recurrence in patients with colonic CD; however, SC was associated with fewer permanent stomas but a higher rate of postoperative complications compared with subtotal colectomy [5]. A different meta-analysis by Tekkis et al. included six studies comparing SC with subtotal or total colectomy and concluded that both approaches were equally effective, although early recurrences were more common in the SC group [26].

A factor potentially influencing recurrence, but inconsistently reported across the included studies, is the performance of extended mesenteric resection. Emerging evidence suggests that mesenteric excision reduces surgical recurrence without increasing postoperative complications [28]. However, as a result of insufficient reporting, this variable could not be evaluated in the present analysis, highlighting a pertinent direction for future research. The higher rates observed in the SC group may reflect that this surgical approach may inadequately address microscopic or subclinical disease present in segments of the colon that were not resected, potentially leading to earlier relapse and symptom recurrence. This pattern was mirrored in the Bayesian analyses: despite wide posterior uncertainty, the posterior distributions consistently suggested a higher probability of surgical recurrence and reoperation after SC. By expressing these effects in probabilistic terms, the Bayesian framework reinforces that the posterior distributions suggest a tendency toward higher recurrence in the SC group, although the probability remains close to equipoise and instead reflects a clinically meaningful trade-off between long-term disease control and stoma avoidance. Nevertheless, the analysis of recurrence remains subject to a specific limitation due to potential underreporting and inconsistent definitions in many of the included studies; in particular, we included studies reporting recurrence episodes even without subsequent surgical reintervention, which may have influenced the accuracy and precision of the recurrence estimates in both the frequentist and Bayesian models.

Regarding functional and safety outcomes, we did not observe significant differences between the groups in terms of permanent stoma formation and overall complications in the frequentist analyses. The absence of a statistically significant difference in permanent stoma formation between SC and EC groups suggests that preserving colonic tissue with segmental resection may not necessarily confer a functional advantage regarding stoma avoidance. Likewise, the similar complication outcomes imply that the extent of surgical resection alone does not markedly affect short-term surgical safety. By contrast, the Bayesian analysis restricted to permanent stoma offers an additional layer of interpretability: despite wide posterior uncertainty, the probability that SC reduces the need for a permanent stoma compared with EC was consistently high across all prior specifications. In a context where traditional hypothesis testing did not demonstrate statistical significance, this probabilistic signal suggests that the apparent neutrality of stoma-related outcomes may, at least in part, reflect limited statistical power rather than a true absence of effect. Taken together, these findings indicate that short-term safety is broadly similar between SC and EC, while the Bayesian estimates raise the possibility of a clinically meaningful advantage of SC with respect to stoma avoidance, which should be further explored in prospective studies explicitly designed and adequately powered for this endpoint.

Finally, the Bayesian framework complemented the frequentist analysis by quantifying the probability of benefit or harm with greater clinical interpretability. Despite wide uncertainty, posterior estimates suggested a higher likelihood of surgical recurrence and reoperation after SC, whereas the probability of reducing permanent stoma was uniformly high across all priors. These findings were robust in sensitivity analyses and provide a probabilistic context that strengthens decision-making in settings where the evidence base remains limited.

This study has several limitations that should be acknowledged. First, most included studies were observational and retrospective in nature, which limits the level of evidence and precludes the establishment of causal relationships. Such designs remain susceptible to confounding factors and selection biases that may have influenced the outcomes. Second, all studies were assessed as having a moderate risk of bias, which further reduces the certainty of the conclusions. Third, substantial heterogeneity was observed across several outcomes, and variations in the definitions of clinical and surgical recurrence may have contributed to this variability. Although statistical methods, sensitivity analyses, and subgroup analyses were applied to address heterogeneity, its persistence indicates that results should be interpreted with caution. Fourth, no statistical adjustment for multiple testing was performed in subgroup analyses, so these findings should be considered exploratory. Fifth, the absence of patient-level data limited the ability to evaluate characteristics that may influence the comparative effectiveness of SC versus EC. Additionally, the reporting of recurrence outcomes was heterogeneous across studies. Only one study provided detailed information on time to recurrence, while the remaining studies reported only the proportion of patients experiencing surgical recurrence during follow-up. Furthermore, detailed information regarding the clinical presentation and diagnostic pathway leading to recurrence was not specified in most studies, preventing a structured comparison of these variables. Finally, the Bayesian analysis, while offering clinically interpretable posterior probabilities, is limited by the small number of studies, the use of aggregated data, and the influence of prior distributions, especially in outcomes with sparse events, which may affect the stability and generalizability of posterior estimates.

## Conclusion

In patients with colonic CD undergoing surgical resection, the frequentist meta-analysis did not demonstrate statistically significant differences between SC and EC in surgical recurrence, reoperation, permanent stoma formation, postoperative morbidity, or mortality. Bayesian modeling suggested higher probabilities of recurrence and reoperation after SC and of a permanent stoma after EC. However, uncertainty remained substantial as a result of the limited number of studies. These findings suggest a potential trade-off between bowel preservation and long-term disease control and highlight the need for well-designed prospective studies with longer follow-up to better define the optimal surgical strategy for colonic CD.

## Supplementary Information

Below is the link to the electronic supplementary material.

Supplementary file1 (DOCX 771 KB)

Supplementary file2 (DOCX 10 KB)

## Data Availability

This systematic review and meta-analysis used publicly available data from the researched databases. No new datasets were generated or analyzed during the current study.

## Abbreviations

CD: Crohn’s disease
CI: Confidence interval
EC: Extended colectomy
IBD: Inflammatory bowel disease
MH: Mantel–Haenszel method
OR: Odds ratio
PRISMA: Preferred Reporting Items for Systematic Reviews and Meta-Analyses
PROSPERO: International Prospective Register of Systematic Reviews
ROBINS-I: Risk of Bias In Non-Randomized Studies of Interventions
SC: Segmental colectomy
