# Supplementary material for: Segmental versus extended colectomy for colonic Crohn’s disease in advanced therapy era: a systematic review and Bayesian meta-analysis
Source: Tech Coloproctol. 2026 Jun 16;30(1):135. doi: 10.1007/s10151-026-03355-z (PMC13506587; doi:10.1007/s10151-026-03355-z)
Supplement: Supplementary file 1 [file 10151_2026_3355_MOESM1_ESM.docx]

**SUPPLEMENTARY MATERIAL 1**

**TITLE:** Segmental versus Extended Colectomy for Colonic Crohn’s Disease in Advanced Therapy Era: A Systematic Review and Bayesian Meta Analysis

**SUPPLEMENTARY TABLES AND FIGURES LEGENDS:**

**Supplementary Table S1.** Preferred Reporting Items for Systematic Reviews and Meta-Analysis (PRISMA) checklist.

**Supplementary Table S2.** List of excluded studies at the full-text screening stage and the reasons for exclusion.

**Supplementary Table S3.** Prior specification table (primary and sensitivity priors) for surgical recurrence.

**Supplementary Table S4.** Model interpretation table (μ, τ, posteriors, clinical probability) for surgical recurrence.

**Supplementary Table S5.** Posterior clinical probabilities for surgical recurrence.

**Supplementary Table S6.** Prior specification table (primary and sensitivity priors) for reoperation.

**Supplementary Table S7.** Model interpretation table (μ, τ, posteriors, clinical probability) for reoperation.

**Supplementary Table S8.** Posterior clinical probabilities for reoperation.

**Supplementary Table S9.** Prior specification table (primary and sensitivity priors) for permanent stoma.

**Supplementary Table S10.** Model interpretation table (μ, τ, posteriors, clinical probability) for permanent stoma.

**Supplementary Table S11.** Posterior clinical probabilities for permanent stoma.

**Supplementary Table S12.** Risk of bias assessment.

**Supplementary Figure S1**.Prior specifications for effect (μ) and heterogeneity (τ) in the Bayesian analysis for surgical recurrence.

**Supplementary Figure S2**.Prior specifications for effect (μ) and heterogeneity (τ) in the Bayesian analysis for reoperation.

**Supplementary Figure S3**.Prior specifications for effect (μ) and heterogeneity (τ) in the Bayesian analysis for permanent stoma.

**Supplementary Figure S4**. Leave-one-out analysis for surgical recurrence. CI: confidence interval; SC: segmental colectomy; EC: extended colectomy; MH: Mantel-Haenszel method; OR: odds ratio.

**Supplementary Figure S5**. Leave-one-out analysis for reoperation. CI: confidence interval; SC: segmental colectomy; EC: extended colectomy; MH: Mantel-Haenszel method; OR: odds ratio.

**Supplementary Figure S6**. Leave-one-out analysis for permanent stoma. CI: confidence interval; SC: segmental colectomy; EC: extended colectomy; MH: Mantel-Haenszel method; OR: odds ratio.

**Supplementary Figure S7**. Leave-one-out analysis for postoperative morbidity. CI: confidence interval; SC: segmental colectomy; EC: extended colectomy; MH: Mantel-Haenszel method; OR: odds ratio.

**Supplementary Table S1.** Preferred Reporting Items for Systematic Reviews and Meta-Analysis (PRISMA) checklist.

| **Section and Topic** | **Item #** | **Checklist item** | **Location where item is reported** |
| --- | --- | --- | --- |
| **TITLE** | | |  |
| Title | 1 | Identify the report as a systematic review. | Page 1 |
| **ABSTRACT** | | |  |
| Abstract | 2 | See the PRISMA 2020 for Abstracts checklist. | - |
| **INTRODUCTION** | | |  |
| Rationale | 3 | Describe the rationale for the review in the context of existing knowledge. | Page 5 |
| Objectives | 4 | Provide an explicit statement of the objective(s) or question(s) the review addresses. | Page 5 |
| **METHODS** | | |  |
| Eligibility criteria | 5 | Specify the inclusion and exclusion criteria for the review and how studies were grouped for the syntheses. | Page 6 |
| Information sources | 6 | Specify all databases, registers, websites, organisations, reference lists and other sources searched or consulted to identify studies. Specify the date when each source was last searched or consulted. | Pages 6-7 |
| Search strategy | 7 | Present the full search strategies for all databases, registers and websites, including any filters and limits used. | Pages 6-7 |
| Selection process | 8 | Specify the methods used to decide whether a study met the inclusion criteria of the review, including how many reviewers screened each record and each report retrieved, whether they worked independently, and if applicable, details of automation tools used in the process. | Pages 6-7 |
| Data collection process | 9 | Specify the methods used to collect data from reports, including how many reviewers collected data from each report, whether they worked independently, any processes for obtaining or confirming data from study investigators, and if applicable, details of automation tools used in the process. | Page 7 |
| Data items | 10a | List and define all outcomes for which data were sought. Specify whether all results that were compatible with each outcome domain in each study were sought (e.g. for all measures, time points, analyses), and if not, the methods used to decide which results to collect. | Page 6 |
|  | 10b | List and define all other variables for which data were sought (e.g. participant and intervention characteristics, funding sources). Describe any assumptions made about any missing or unclear information. | Page 8 |
| Study risk of bias assessment | 11 | Specify the methods used to assess risk of bias in the included studies, including details of the tool(s) used, how many reviewers assessed each study and whether they worked independently, and if applicable, details of automation tools used in the process. | Page 7 |
| Effect measures | 12 | Specify for each outcome the effect measure(s) (e.g. risk ratio, mean difference) used in the synthesis or presentation of results. | Pages 7-9 |
| Synthesis methods | 13a | Describe the processes used to decide which studies were eligible for each synthesis (e.g. tabulating the study intervention characteristics and comparing against the planned groups for each synthesis (item #5)). | Pages 7-9 |
|  | 13b | Describe any methods required to prepare the data for presentation or synthesis, such as handling of missing summary statistics, or data conversions. | Pages 7-9 |
|  | 13c | Describe any methods used to tabulate or visually display results of individual studies and syntheses. | Pages 7-9 |
|  | 13d | Describe any methods used to synthesize results and provide a rationale for the choice(s). If meta-analysis was performed, describe the model(s), method(s) to identify the presence and extent of statistical heterogeneity, and software package(s) used. | Pages 7-9 |
|  | 13e | Describe any methods used to explore possible causes of heterogeneity among study results (e.g. subgroup analysis, meta-regression). | Pages 7-9 |
|  | 13f | Describe any sensitivity analyses conducted to assess robustness of the synthesized results. | Pages 7-9 |
| Reporting bias assessment | 14 | Describe any methods used to assess risk of bias due to missing results in a synthesis (arising from reporting biases). | Page 7 |
| Certainty assessment | 15 | Describe any methods used to assess certainty (or confidence) in the body of evidence for an outcome. | Page 7 |
| **RESULTS** | | |  |
| Study selection | 16a | Describe the results of the search and selection process, from the number of records identified in the search to the number of studies included in the review, ideally using a flow diagram. | Page 9 |
|  | 16b | Cite studies that might appear to meet the inclusion criteria, but which were excluded, and explain why they were excluded. | Figure 1 |
| Study characteristics | 17 | Cite each included study and present its characteristics. | Table 1 |
| Risk of bias in studies | 18 | Present assessments of risk of bias for each included study. | Supplementary Table S12 |
| Results of individual studies | 19 | For all outcomes, present, for each study: (a) summary statistics for each group (where appropriate) and (b) an effect estimate and its precision (e.g. confidence/credible interval), ideally using structured tables or plots. | Pages 10-12 |
| Results of syntheses | 20a | For each synthesis, briefly summarise the characteristics and risk of bias among contributing studies. | Pages 10-12 |
|  | 20b | Present results of all statistical syntheses conducted. If meta-analysis was done, present for each the summary estimate and its precision (e.g. confidence/credible interval) and measures of statistical heterogeneity. If comparing groups, describe the direction of the effect. | Pages 10-12 |
|  | 20c | Present results of all investigations of possible causes of heterogeneity among study results. | Pages 10-12 |
|  | 20d | Present results of all sensitivity analyses conducted to assess the robustness of the synthesized results. | Pages 10-12 |
| Reporting biases | 21 | Present assessments of risk of bias due to missing results (arising from reporting biases) for each synthesis assessed. | NA |
| Certainty of evidence | 22 | Present assessments of certainty (or confidence) in the body of evidence for each outcome assessed. | NA |
| **DISCUSSION** | | |  |
| Discussion | 23a | Provide a general interpretation of the results in the context of other evidence. | Pages 12-15 |
|  | 23b | Discuss any limitations of the evidence included in the review. | Pages 12-15 |
|  | 23c | Discuss any limitations of the review processes used. | Pages 12-15 |
|  | 23d | Discuss implications of the results for practice, policy, and future research. | Pages 12-15 |
| **OTHER INFORMATION** | | |  |
| Registration and protocol | 24a | Provide registration information for the review, including register name and registration number, or state that the review was not registered. | Page 6 |
|  | 24b | Indicate where the review protocol can be accessed, or state that a protocol was not prepared. | Page 6 |
|  | 24c | Describe and explain any amendments to information provided at registration or in the protocol. | Page 6 |
| Support | 25 | Describe sources of financial or non-financial support for the review, and the role  of the funders or sponsors in the review. | Page 18 |
| Competing interests | 26 | Declare any competing interests of review authors. | Page 2 |
| Availability of data, code and other materials | 27 | Report which of the following are publicly available and where they can be found: template data collection forms; data extracted from included studies; data used for all analyses; analytic code; any other materials used in the review. | Page 2 |

*From:*  Page MJ, McKenzie JE, Bossuyt PM, Boutron I, Hoffmann TC, Mulrow CD, et al. The PRISMA 2020 statement: an updated guideline for reporting systematic reviews. BMJ 2021;372:n71. doi: 10.1136/bmj.n71

**Supplementary Table S2.** List of excluded studies at the full-text screening stage and the reasons for exclusion.

| **First author, year [ref]** | **Reason for exclusion** |
| --- | --- |
| Cherubini, 2022 [1] | Conference abstract |
| Frontali, 2020 [2] | Conference abstract |
| Haassan, 2016 [3] | Conference abstract |
| Tulchinsky, 2015 [4] | Conference abstract |
| Maser, 2012 [5] | Conference abstract |
| Canelas, 2011 [6] | Conference abstract |
| Bogach, 2020 [7] | Overlapping population |
| Kiran, 2012 [8] | No comparison between SC and EC |
| Imigo, 2018 [9] | No comparison between SC and EC |
| Martel, 2002 [10] | No comparison between SC and EC |
| Allan, 1989 [11] | Studies before biologic therapy conception |
| Andersson, 2002 [12] | Studies before biologic therapy conception |
| Andrews, 1989 [13] | Studies before biologic therapy conception |
| Bernell, 2001 [14] | Studies before biologic therapy conception |
| Fichera, 2005 [15] | Studies before biologic therapy conception |
| Longo, 1988 [16] | Studies before biologic therapy conception |
| Prabhakar, 1997 [17] | Studies before biologic therapy conception |
| Bogach, 2021 [18] | No outcomes of interest |

1. Cherubini, V., Mineccia, M., Vercellone, B., Palisi, M., Massucco, P., & Ferrero, A. (2022). Surgical recurrence in Crohn colitis. *Techniques in Coloproctology, 26*, 503–512. Presented at the 9th National Congress of The Italian Society of Colorectal Surgery, Naples, October 24–26, 2021.<https://doi.org/10.1007/s10151-022-02606-z>
2. Frontali, A., Chierici, A., Prost-A-La Denise, J., Treton, X., Maggiori, L., Bouhnik, Y., & Panis, Y. (2020). Segmental colectomy versus extended colectomy for colonic Crohn’s disease: Results from 112 consecutive patients. *Techniques in Coloproctology, 24*, 337–388. Presented at the 8th National Congress of The Italian Society of Colorectal Surgery, Biella, November 11–13, 2019.<https://doi.org/10.1007/s10151-020-02154-4>
3. Haassan, T., Ozuner, G., Aytac, E., & Remzi, F. (2016). Crohn's colitis: What is the preferred management? Segmental, subtotal or total colectomy: An analysis of outcomes and recurrence. *Diseases of the Colon & Rectum, 59*(5), e49-e400. The American Society of Colon and Rectal Surgeons.<https://doi.org/10.1097/01.dcr.0000482708.50838.af>
4. Tulchinsky, H., Bendersky, M., Yanai, H., Klausner, J., & Dotan, I. (2015). Comparison between total abdominal colectomy and segmental colectomy in Crohn’s colitis patients: Recurrence and re-resection rates. A retrospective cohort study. *Journal of Crohn's and Colitis, 9*(Suppl_1), S391–S392.<https://doi.org/10.1093/ecco-jcc/jju027.739>
5. Maser, A. E., Kruse, D., Harpaz, N., Powers, S. E., Sachar, D. B., & Bauer, J. J. (2012). Recurrent colon cancer or dysplasia after segmental resection in Crohn’s colitis. *Gastroenterology, 142*(5 Suppl 1), S189. Presented at Digestive Disease Week 2012 (DDW 2012), San Diego, CA, May 19–22, 2012.
6. Canelas, A., Bun, M., Grzona, E., Goldaracena, N., Laporte, M., & Roholtz, N. (2011). Laparoscopic total colectomy: Feasibility and results. *Surgical Endoscopy and Other Interventional Techniques, 25*(Suppl 1), S271. Presented at the 2011 Scientific Session of the Society of American Gastrointestinal and Endoscopic Surgeons (SAGES), San Antonio, TX, March 30–April 2, 2011.<https://doi.org/10.1007/s00464-011-1598-0>
7. Bogach, J., Pond, G., Eskicioglu, C., & Seow, H. (2020). The extent of resection and survival in inflammatory bowel disease-associated colorectal cancer: A population-based study. *Annals of Surgical Oncology, 27*(Suppl 1), 1–230. Presented at the Society of Surgical Oncology (SSO) 2020 – International Conference on Surgical Cancer Care.<https://doi.org/10.1245/s10434-020-08278-z>
8. Kiran, R. P., Nisar, P. J., Goldblum, J. R., Fazio, V. W., Remzi, F. H., Shen, B., & Lavery, I. C. (2012). Dysplasia associated with Crohn's colitis: Segmental colectomy or more extended resection? *Annals of Surgery, 256*(2), 221–226. https://doi.org/10.1097/SLA.0b013e31825f0709
9. Imigo, G. F., Molina, P. M. E., Álvarez-Lobos, M., Quintana, V. C., Klaassen, L. J., Torres, M. J., Duarte, G. I., Bellolio, R. F., & Zúñiga, D. Á. (2018). Colitis de Crohn: Resultados del tratamiento quirúrgico y evolución alejada [Outcome of surgery for Crohn's colitis. Review of 28 cases]. *Revista Médica de Chile, 146*(2), 183–189. https://doi.org/10.4067/s0034-98872018000200183
10. Martel, P., Betton, P. O., Gallot, D. M., & Malafosse, M. M. (2002). Crohn's colitis: Experience with segmental resections; Results in a series of 84 patients. *Journal of the American College of Surgeons, 194*(4), 448–453. <https://doi.org/10.1016/S1072-7515(02)01122-5>
11. Allan A, Andrews H, Hilton CJ, Keighley MR, Allan RN, Alexander-Williams J. Segmental colonic resection is an appropriate operation for short skip lesions due to Crohn's disease in the colon. World J Surg. 1989 Sep-Oct;13(5):611-4; discussion 615-6. doi: 10.1007/BF01658882. PMID: 2815805.
12. Andersson P, Olaison G, Hallböök O, Sjödahl R. Segmental resection or subtotal colectomy in Crohn's colitis? Dis Colon Rectum. 2002 Jan;45(1):47-53. doi: 10.1007/s10350-004-6113-4. PMID: 11786764.
13. Andrews HA, Lewis P, Allan RN. Prognosis after surgery for colonic Crohn's disease. Br J Surg. 1989 Nov;76(11):1184-90. doi: 10.1002/bjs.1800761123. PMID: 2597977.
14. Bernell O, Lapidus A, Hellers G. Recurrence after colectomy in Crohn's colitis. Dis Colon Rectum. 2001 May;44(5):647-54; discussion 654. doi: 10.1007/BF02234559. PMID: 11357022.
15. Fichera A, McCormack R, Rubin MA, Hurst RD, Michelassi F. Long-term outcome of surgically treated Crohn's colitis: a prospective study. Dis Colon Rectum. 2005 May;48(5):963-9. doi: 10.1007/s10350-004-0906-3. PMID: 15785882.
16. Longo WE, Ballantyne GH, Cahow CE. Treatment of Crohn's colitis. Segmental or total colectomy? Arch Surg. 1988 May;123(5):588-90. doi: 10.1001/archsurg.1988.01400290070011. PMID: 3358685.
17. Prabhakar LP, Laramee C, Nelson H, Dozois RR. Avoiding a stoma: role for segmental or abdominal colectomy in Crohn's colitis. Dis Colon Rectum. 1997 Jan;40(1):71-8. doi: 10.1007/BF02055685. PMID: 9102265.
18. Bogach J, Pond G, Eskicioglu C, Simunovic M, Seow H. Extent of Surgical Resection in Inflammatory Bowel Disease Associated Colorectal Cancer: a Population-Based Study. J Gastrointest Surg. 2021 Oct;25(10):2610-2618. doi: 10.1007/s11605-021-04913-6. Epub 2021 Feb 8. PMID: 33559097.

**Supplementary Table S3.** Prior specification table (primary and sensitivity priors) for surgical recurrence.

| **Outcome / Scale** | **Primary Prior** | **Sensitivity Prior 1** | **Sensitivity Prior 2** | **Justification** |
| --- | --- | --- | --- | --- |
| Surgical Recurrence (log-OR) | μ ~ Normal(0, 2.7²) τ ~ Log-Normal(Turner) [meanlog = −0.845, sdlog = 0.84] | μ ~ Normal(0, 2.7²) τ ~ Half-Normal(0.5) | μ ~ Normal(0, 2.7²) τ ~ Half-Normal(1.5) | Primary prior uses Turner’s empirical distribution for surgical/device outcomes as an informative prior for τ. Sensitivity priors relax this assumption using weakly informative Half-Normal(0.5) and vague Half-Normal(1.5) heterogeneity priors. |

**Supplementary Table S4.** Model interpretation table (μ, τ, posteriors, clinical probability) for surgical recurrence.

| **Model** | **Prior for μ (Effect Size)** | **Interpretation of μ Prior** | **Prior for τ (Heterogeneity)** | **Interpretation of τ Prior** | **Posterior OR (mean)** | **95 % CrI** | **P(OR ≤ 1)** | **Interpretation** |
| --- | --- | --- | --- | --- | --- | --- | --- | --- |
| **Primary (Turner)** | Normal(0, 2.7²) | Vague prior; does not constrain direction of effect | Log-Normal (Turner) | Informative, grounded in empirical distributions for surgical/device outcomes | 1.10 | 0.48 to 2.75 | 42.5 % | Posterior slightly favors lower recurrence with SC, but credible intervals remain wide and clinically important benefit or harm cannot be excluded |
| **Sensitivity 1 (HN 0.5)** | Normal(0, 2.7²) | Same vague prior | Half-Normal(0.5) | Weakly informative heterogeneity prior | 01.08 | 0.52 to 2.45 | 44.1 % | Similar posterior distribution, indicating robustness to an alternative heterogeneity prior |
| **Sensitivity 2 (HN 1.5)** | Normal(0, 2.7²) | Same vague prior | Half-Normal(1.5) | Very vague prior allowing larger heterogeneity | 1.14 | 0.34 to 4.29 | 41.1 % | Wider credible interval reflecting greater uncertainty under very vague heterogeneity assumptions |
| **Historical μ (Angriman)** | Normal(-0.65, 0.20²) | Prior centered on historical data favoring lower recurrence with SC | Log-Normal (Turner) | Same informative surgical τ prior | 0.62 | 0.42 to 0.89 | 99.7 % | Incorporating historical information shifts the posterior toward lower recurrence with SC |

**Supplementary Table S5.** Posterior clinical probabilities for surgical recurrence.

| **Posterior Probability** | **Value** | **Clinical Interpretation** |
| --- | --- | --- |
| **P(OR ≤ 1.0)** | 42.5% | Probability that SC has lower recurrence than EC |
| **P(OR > 1.0)** | 57.5% | Probability that EC has lower recurrence (higher recurrence with SC) |
| **P(OR ≤ 0.80)** | 20.0% | Probability of a clinically meaningful reduction in recurrence with SC |
| **P(OR ≥ 1.25)** | 33.9% | Probability of clinically relevant higher recurrence with SC |

| **Posterior Probability** | **Classification** |
| --- | --- |
| **< 0.20** | **Evidence against (weak / minimal)** |
| **0.20 – 0.40** | **Weak evidence** |
| **0.40 – 0.60** | **No evidence (equivocal)** |
| **0.60 – 0.80** | **Moderate evidence** |
| **> 0.80** | **Strong evidence** |
| **> 0.95** | **Very strong / decisive** |

**Supplementary Table S6.** Prior specification table (primary and sensitivity priors) for reoperation.

| **Outcome / Scale** | **Primary Prior** | **Sensitivity Prior 1** | **Sensitivity Prior 2** | **Justification** |
| --- | --- | --- | --- | --- |
| μ (effect size) | Normal(0, 2.7²) | Normal(0, 2.7²) | Normal(0, 2.7²) | Vague prior commonly used for treatment effects in Bayesian meta-analysis of surgical trials |
| τ (heterogeneity) | Log-Normal (Turner empirical prior) | Half-Normal(0.5) | Half-Normal(1.5) | Turner prior reflects empirical heterogeneity distributions observed in surgical/device outcomes; half-normal priors evaluate robustness to more restrictive or more permissive heterogeneity assumptions |

**Supplementary Table S7.** Model interpretation table (μ, τ, posteriors, clinical probability) for reoperation.

| **Model** | **Prior for μ** | **Prior for τ** | **Posterior OR** | **95% CrI** | **P(OR ≤1)** | **Interpretation** |
| --- | --- | --- | --- | --- | --- | --- |
| Primary (Turner) | Normal(0,2.7²) | Log-Normal (Turner) | 1.42 | 0.68–3.12 | 16.1% | Posterior distribution favors EC, with a low probability that SC reduces reoperation. Credible intervals remain wide and include the null. |
| Sensitivity 1 | Normal(0,2.7²) | Half-Normal(0.5) | 1.41 | 0.71–2.93 | 15.7% | Posterior estimate remains similar, indicating robustness to a more restrictive heterogeneity prior. |
| Sensitivity 2 | Normal(0,2.7²) | Half-Normal(1.5) | 1.47 | 0.58–3.96 | 18.0% | Wider credible interval reflecting greater uncertainty under more permissive heterogeneity assumptions. |

**Supplementary Table S8.** Posterior clinical probabilities for reoperation.

| **Posterior Probability** | **Value** | **Clinical Interpretation** |
| --- | --- | --- |
| **P(OR ≤ 1.0)** | 16.1% | Probability that SC has lower reoperation rate than EC |
| **P(OR > 1.0)** | 83.9% | Probability that reoperation is higher with SC than EC |
| **P(OR ≤ 0.75)** | 3.7% | Probability of a clinically meaningful benefit of SC |
| **P(OR ≥ 1.5)** | 41.6% | Probability of substantially worse reoperation outcomes with SC |

| **Posterior Probability** | **Classification** |
| --- | --- |
| **< 0.20** | **Evidence against (weak / minimal)** |
| **0.20 – 0.40** | **Weak evidence** |
| **0.40 – 0.60** | **No evidence (equivocal)** |
| **0.60 – 0.80** | **Moderate evidence** |
| **> 0.80** | **Strong evidence** |
| **> 0.95** | **Very strong / decisive** |

**Supplementary Table S9.** Prior specification table (primary and sensitivity priors) for permanent stoma.

| **Component** | **Primary Prior** | **Sensitivity 1** | **Sensitivity 2** | **Historical μ Test** | **Justification** |
| --- | --- | --- | --- | --- | --- |
| **μ (effect)** | Normal(0, 2.7²) | Normal(0, 2.7²) | Normal(0, 2.7²) | Normal(–0.65, 0.20²) | Vague prior (primary); historical prior from Angriman for comparison |
| **τ (heterogeneity)** | Turner Log-Normal (–0.845, 0.84) | Half-Normal(0.5) | Half-Normal(1.5) | Turner Log-Normal | Turner recommended for surgical/device outcomes; half-normal for robustness |

**Supplementary Table S10.** Model interpretation table (μ, τ, posteriors, clinical probability) for permanent stoma.

| **Model** | **μ Prior** | **τ Prior** | **Posterior OR** | **95 % CrI** | **P(OR ≤ 1)** | **Interpretation** |
| --- | --- | --- | --- | --- | --- | --- |
| **Primary** | Normal(0, 2.7²) | Turner | 0.23 | 0.054–1.14 | 96.4 % | Strong evidence SC reduces permanent stoma |
| **Sensitivity 1** | Normal(0, 2.7²) | HN(0.5) | 0.22 | 0.073–0.69 | 99.2 % | Very strong evidence; low heterogeneity assumption makes effect more precise |
| **Sensitivity 2** | Normal(0, 2.7²) | HN(1.5) | 0.26 | 0.041–1.86 | 92.5 % | Strong evidence even with permissive heterogeneity |
| **Historical μ (Angriman)** | Normal(–0.65, 0.20²) | Turner | 0.49 | 0.33–0.72 | 100 % | Fully consistent with prior evidence; confirms effect |

**Supplementary Table S11.** Posterior clinical probabilities for permanent stoma.

| **Posterior Probability** | **Value** | **Clinical Interpretation** |
| --- | --- | --- |
| **P(OR ≤ 1.0)** | **96.4%** | Strong evidence of a lower stoma risk with SC |
| **P(OR > 1.0)** | 3.6 **%** | No relevant evidence in favor of EC |
| **P(OR ≤ 0.75)** | **93.8 %** | A clinically relevant benefit is very likely |
| **P(OR ≤ 0.5)** | **86.6 %** | A large clinical benefit of SC is highly probable |

| **Posterior Probability** | **Classification** |
| --- | --- |
| **< 0.20** | **Evidence against (weak / minimal)** |
| **0.20 – 0.40** | **Weak evidence** |
| **0.40 – 0.60** | **No evidence (equivocal)** |
| **0.60 – 0.80** | **Moderate evidence** |
| **> 0.80** | **Strong evidence** |
| **> 0.95** | **Very strong / decisive** |

**Supplementary Table S12.** Risk of bias assessment.

| First author, year | Domain 1 | Domain 2 | Domain 3 | Domain 4 | Domain 5 | Domain 6 | Domain 7 |
| --- | --- | --- | --- | --- | --- | --- | --- |
| Celentano, 2021 | Moderate | Low | Moderate | Moderate | Moderate | Moderate | Low |
| Kiran, 2011 | Moderate | Low | Moderate | Moderate | Moderate | Moderate | Low |
| Lee 2016 | Moderate | Low | Moderate | Moderate | Low | Moderate | Low |
| Pellino, 2022 | Moderate | Low | Moderate | Moderate | Moderate | Moderate | Low |
| Sensi, 2022 | Moderate | Low | Moderate | Moderate | Moderate | Moderate | Low |

Domains:

D1: Bias due to confounding.

D2: Bias in the classification of interventions.

D3: Bias in the selection of participants into the study or analysis.

D4: Bias due to deviations from intended interventions.

D5: Bias due to missing data.

D6: Bias in the measurement of outcomes.

D7: Bias in the selection of reported results.

**Supplementary Figure S1**.Prior specifications for effect (μ) and heterogeneity (τ) in the Bayesian analysis for surgical recurrence.

**Supplementary Figure S2**.Prior specifications for effect (μ) and heterogeneity (τ) in the Bayesian analysis for reoperation.

**Supplementary Figure S3**.Prior specifications for effect (μ) and heterogeneity (τ) in the Bayesian analysis for permanent stoma.

**Supplementary Figure S4**. Leave-one-out analysis for surgical recurrence. CI: confidence interval; SC: segmental colectomy; EC: extended colectomy; MH: Mantel-Haenszel method; OR: odds ratio.

**Supplementary Figure S5**. Leave-one-out analysis for reoperation. CI: confidence interval; SC: segmental colectomy; EC: extended colectomy; MH: Mantel-Haenszel method; OR: odds ratio.

**Supplementary Figure S6**. Leave-one-out analysis for permanent stoma. CI: confidence interval; SC: segmental colectomy; EC: extended colectomy; MH: Mantel-Haenszel method; OR: odds ratio.

**Supplementary Figure S7**. Leave-one-out analysis for overall complications. CI: confidence interval; SC: segmental colectomy; EC: extended colectomy; MH: Mantel-Haenszel method; OR: odds ratio.
