# Supplementary material for: Segmental versus extended colectomy for colonic Crohn’s disease in advanced therapy era: a systematic review and Bayesian meta-analysis
Source: Tech Coloproctol. 2026 Jun 16;30(1):135. doi: 10.1007/s10151-026-03355-z (PMC13506587; doi:10.1007/s10151-026-03355-z)
Supplement: Supplementary file 2 [file 10151_2026_3355_MOESM2_ESM.docx]

**SUPPLEMENTARY MATERIAL 2**

**TITLE:** Segmental versus Extended Colectomy for Colonic Crohn’s Disease in Advanced Therapy Era: A Systematic Review and Bayesian Meta Analysis

**SUPPLEMENTARY TABLES LEGENDS:**

**Supplementary Table S1.** Raw data for surgical recurrence. Abbreviations: e.e., events in the segmental colectomy group; n.e., number of patients in the segmental colectomy group; e.c., events in the extended colectomy group; n.c., number of patients in the extended colectomy group.

**Supplementary Table S2.** Raw data for reoperation. Abbreviations: e.e., events in the segmental colectomy group; n.e., number of patients in the segmental colectomy group; e.c., events in the extended colectomy group; n.c., number of patients in the extended colectomy group.

**Supplementary Table S3.** Raw data for permanent stoma. Abbreviations: e.e., events in the segmental colectomy group; n.e., number of patients in the segmental colectomy group; e.c., events in the extended colectomy group; n.c., number of patients in the extended colectomy group.

**Supplementary Table S4.** Raw data for overall complications. Abbreviations: e.e., events in the segmental colectomy group; n.e., number of patients in the segmental colectomy group; e.c., events in the extended colectomy group; n.c., number of patients in the extended colectomy group.

**Supplementary Table S5.** Raw data for postoperative mortality. Abbreviations: e.e., events in the segmental colectomy group; n.e., number of patients in the segmental colectomy group; e.c., events in the extended colectomy group; n.c., number of patients in the extended colectomy group.

**Supplementary Table S1.** Raw data for surgical recurrence. Abbreviations: e.e., events in the segmental colectomy group; n.e., number of patients in the segmental colectomy group; e.c., events in the extended colectomy group; n.c., number of patients in the extended colectomy group.

| Study | e.e | n.e | e.c | n.c |
| --- | --- | --- | --- | --- |
| Kiran 2011 | 13 | 49 | 8 | 59 |
| Lee 2016 | 26 | 71 | 13 | 45 |
| Pellino 2022 | 43 | 285 | 88 | 402 |

**Supplementary Table S2.** Raw data for reoperation. Abbreviations: e.e., events in the segmental colectomy group; n.e., number of patients in the segmental colectomy group; e.c., events in the extended colectomy group; n.c., number of patients in the extended colectomy group.

| Study | e.e | n.e | e.c | n.c |
| --- | --- | --- | --- | --- |
| Calentano 2021 | 4 | 30 | 3 | 67 |
| Kiran 2011 | 22 | 49 | 15 | 59 |
| Lee 2016 | 35 | 71 | 18 | 45 |
| Pellino 2022 | 43 | 285 | 88 | 402 |
| Sensi 2022 | 5 | 66 | 2 | 33 |

**Supplementary Table S3.** Raw data for permanent stoma. Abbreviations: e.e., events in the segmental colectomy group; n.e., number of patients in the segmental colectomy group; e.c., events in the extended colectomy group; n.c., number of patients in the extended colectomy group.

| Study | e.e | n.e | e.c | n.c |
| --- | --- | --- | --- | --- |
| Kiran 2011 | 6 | 49 | 5 | 59 |
| Lee 2016 | 4 | 71 | 23 | 45 |
| Pellino 2022 | 23 | 285 | 158 | 402 |

**Supplementary Table S4.** Raw data for overall complications. Abbreviations: e.e., events in the segmental colectomy group; n.e., number of patients in the segmental colectomy group; e.c., events in the extended colectomy group; n.c., number of patients in the extended colectomy group.

| Study | e.e | n.e | e.c | n.c |
| --- | --- | --- | --- | --- |
| Calentano 2021 | 11 | 30 | 28 | 67 |
| Kiran 2011 | 32 | 49 | 32 | 59 |
| Lee 2016 | 17 | 71 | 25 | 45 |
| Pellino 2022 | 73 | 285 | 127 | 402 |
| Sensi 2022 | 5 | 66 | 2 | 33 |

**Supplementary Table S5.** Raw data for postoperative mortality. Abbreviations: e.e., events in the segmental colectomy group; n.e., number of patients in the segmental colectomy group; e.c., events in the extended colectomy group; n.c., number of patients in the extended colectomy group.

| Study | e.e | n.e | e.c | n.c |
| --- | --- | --- | --- | --- |
| Kiran 2011 | 0 | 49 | 0 | 59 |
| Pellino 2022 | 1 | 285 | 5 | 402 |
| Sensi 2022 | 0 | 66 | 0 | 33 |
